# Supplementary material for: COVID-19 and HIV: Clinical Outcomes among Hospitalized Patients in the United States
Source: Biomedicines. 2023 Jul 5;11(7):1904. doi: 10.3390/biomedicines11071904 (PMC10377261; doi:10.3390/biomedicines11071904)
Supplement: Supplementary file 1 [file biomedicines-11-01904-s001.zip › biomedicines-2457838-supplementary.pdf]

Supplementary Materials

Table S1. ICD10 Clinical Modification Codes.

Disease/Procedure	ICD Codes
Covid 19	U071, U00, U49, U50, U85, J1282
HIV	B20, Z21
Vasopressor use	3E030XZ,3E033XZ,3E040XZ,3E043XZ,3E050XZ,3E053XZ,3E060XZ,3E063XZ
Mechanical Circulatory Support	5A02110, 5A02210, 5A0211D, 02HA3RZ, 5A02116, 5A0221D, 5A1522F, 5A1522G, 5A1522H, 5A15A2F, 5A15A2G, 5A15A2H, 5A15223
Mechanical ventilation invasive	0BH17EZ, 0BH18EZ, 5A1935Z, 5A0945Z, 5A0955Z
Non-invasive mechanical ventilation	5A09457, 5A09458, 5A09358, 5A09557, 5A09558, 5A09357
Sudden cardiac arrest	I462, I468, I469
Hemodialysis	5A1D70Z,5A1D90Z,5A1D80Z,5A1D00Z,5A1D60Z
Cardiogenic Shock	R570
CKD	N181, N182, N1830, N1831, N1832, N184, N185, N189
AKI	N170,N171,N172,N178,N179, N990
Acute liver failure	K7200, K7201, K712
VTE	I82210,I82220,I82290,I82401,I82402,I82403,I82409,I82411, I82412,I82413,I82419,I82421,I82422,I82423,I82429,I82431,I82432, I82433,I82439,I82441,I82442,I82443,I82449,I82451,I82452, I82453,I82459,I82461,I82462,I82463,I82469,I82491,I82492,I82493, I82499,I824Y1,I824Y2,I824Y3,I824Y9,I824Z1,I824Z2,I824Z3,I824Z9, I82601,I82602,I82603,I82609,I82611,I82612,I82613,I82619,I82621, I82622,I82623,I82629,I82890,I8290,I82A11,I82A12,I82A13,I82A19, I82B11,I82B12,I82B13,I82B19,I82C11,I82C12,I82C13,I82C19,I1260, I2601,I2602,I2690,I2692,I2693,I2694,I2699,I2609
Smoking	F17, F172, F1720, F17200, F17201, F17203, F17208, F17209, F1721, F17210, F17211, F17213, F17218, F17219, F1722, F17220, F17221, F17223, F17228, F17229, F1729, F17290, F17291, F17293, F17298, F17299, Z87891
Hx of PCI	Z9861, Z9861
Hx of CABG	Z951
Previous MI	I252
CAD	I2510, I25111, I25118, I25119, I252, I253, I254, I2541, I2542, I255, I256, I257, I2570, I25700, I25701, I25708, I25709, I2571, I25710, I25711, I25718, I25719, I2572, I25720, I25721, I25728, I25729, I2573, I25730, I25731, I25738, I25739, I2575, I25750, I25751, I25758, I25759, I2576, I25760, I25761, I25768, I25769
Chronic Pulmonary Disease	Elixhauser comorbidities index
Diabetes (2 types)	Elixhauser comorbidities index
AIDS	Elixhauser comorbidities index
Hypothyroidism	Elixhauser comorbidities index
Autoimmune	Elixhauser comorbidities index
Dementia	Elixhauser comorbidities index
Depression	Elixhauser comorbidities index
Lymphoma	Elixhauser comorbidities index

Leukemia	Elixhauser comorbidities index
Metastatic Cancer	Elixhauser comorbidities index
Solid Tumor Without Metastasis (2 types)	Elixhauser comorbidities index

Obesity	Elixhauser comorbidities index
Drug Abuse	Elixhauser comorbidities index
Hypertension (2 types)	Elixhauser comorbidities index
PAD Peripheral vascular disease	Elixhauser comorbidities index
Alcohol	Elixhauser comorbidities index
Cerebrovascular accident	I6000, I6001, I6002, I6010, I6011, I6012, I602, I6020, I6021, I6022, I6030, I6031, I6032, I604, I6050, I6051, I6052, I606, I607, I608, I609, I610, I611, I612, I613, I614, I615, I616, I618, I619, I6200, I6201, I6202, I6203, I621, I629, I6300, I63011, I63012, I63013, I63019, I6302, I63031, I63032, I63033, I63039, I6309, I6310, I63111, I63112, I63113, I63119, I6312, I63131, I63132, I63133, I63139, I6319, I6320, I63211, I63212, I63213, I63219, I6322, I63231, I63232, I63233, I63239, I6329, I6330, I63311, I63312, I63313, I63319, I63321, I63322, I63323, I63329, I63331, I63332, I63333, I63339, I63341, I63342, I63343, I63349, I6339, I6340, I63411, I63412, I63413, I63419, I63421, I63422, I63423, I63429, I63431, I63432, I63433, I63439, I63441, I63442, I63443, I63449, I6349, I6350, I63511, I63512, I63513, I63519, I63521, I63522, I63523, I63529, I63531, I63532, I63533, I63539, I63541, I63542, I63543, I63549, I6359, I636, I638, I6381, I6389, I639

Table S2. Propensity Matched: Baseline Patients Characteristics 1:1 Propensity matched variables: Age, race, income and insurance status, sex.

**Characteristics**	**COVID and HIV−**	**COVID and HIV+**	**P value**
	%	%	
n = 15,880	7940 (50 %)	7940 (50 %)	--
**Gender (%)**	%	%	0.234
Female	29.97	31.92	
Male	70.02	68.07	
**Mean Age Years (SD)**	**SD**	**SD**	
Female	54.23 (15.67)	54.32 (13.50)	
Male	54.8 (14.58)	54.35 (3.55)	
**AGE Groups (%)**	%	%	<0.001
18-29	6.61	5.16	
30-49	27.52	28.09	
50-69	50.06	55.23	
>=70	15.81	11.52	
**RACE (%)**	%	%	0.961
Asian or Pacific	1.20	1.32	
Black	56.30	55.86	
Hispanic	17.76	18.83	
Native American	0.57	0.44	

Other	4.60	4.41	
White	19.58	19.14	
**MEDIAN HOUSEHOLD INCOME (%)**	%	%	0.731
<= 49,999	49.43	48.11	
50k-64,999	25.56	25.38	
65k-85,999	15.36	15.81	
>=86k	9.65	10.71	
**INSURANCE STATUS (%)**	%	%	0.938
Medicaid	24.69	26.20	
Medicare	38.73	37.91	
No charge	0.44	0.44	
Other	5.04	5.10	
Private Insurance	25.76	24.75	
Self-pay	5.35	5.60	
**HOSPITAL DIVISION (%)**	%	%	<0.001
East North Central	17.19	12.47	
East South Central	4.35	6.42	
Middle Atlantic	33.12	10.71	
Mountain	2.14	4.53	
New England	10.96	6.05	
Pacific	3.46	5.29	
South Atlantic	17.25	38.35	
West North Central	4.09	1.64	
West South Central	7.43	14.55	
**HOSPITAL BEDSIZE (%)**	%	%	0.280
Large	47.54	49.81	
Medium	28.72	28.65	
Small	23.74	21.54	
**HOSPITAL TEACHING STATUS (%)**	%	%	<0.001
Rural	5.29	3.15	
Urban nonteaching	10.01	13.85	
Urban teaching	84.70	83.00	
**COMORBIDITIES (%)**	%	%	
CAD	14.10	12.40	0.157
MI	3.77	3.46	0.634
HTN	62.34	60.51	0.290
Diabetes (2)	38.66	37.46	0.487
Cancer (5)	5.22	5.91	0.394
Obesity	21.78	21.59	0.897
Drug Abuse	6.80	7.68	0.337
Smoking	30.47	33.81	0.044
Alcohol	3.84	3.52	0.637
Chronic Pulmonary Disease	23.23	24.43	0.428
Peripheral Vascular Disease	2.45	2.77	0.578
CKD	10.01	13.16	0.005
Hypothyroidism	4.65	5.60	0.227
Autoimmune	1.70	1.57	0.779
Depression	12.97	13.41	0.713
Dementia	4.84	6.17	0.102

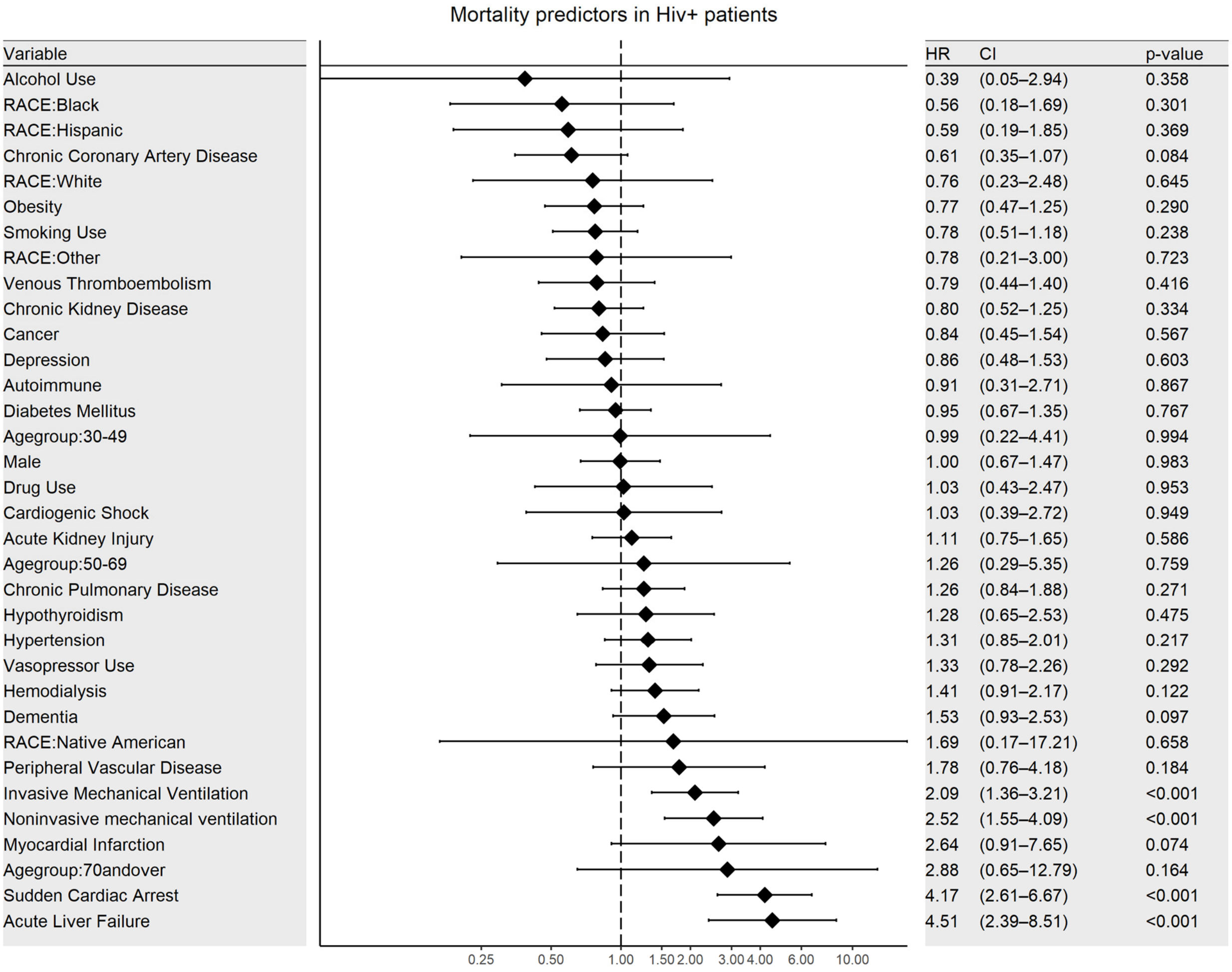

Figure S1. Mortality pedictors in HIV+ patients.
